# Avian influenza, new aspects of an old threat

**DOI:** 10.2807/1560-7917.ES.2023.28.19.2300227

**Published:** 2023-05-11

**Authors:** Cornelia Adlhoch, Francesca Baldinelli

**Affiliations:** 1 European Centre for Disease Prevention and Control (ECDC), Stockholm, Sweden; 2 European Food Safety Authority (EFSA), Parma, Italy

**Keywords:** avian influenza, threat, Europe

Since early 2020, while the worldwide focus was on the COVID-19 pandemic, highly pathogenic avian influenza (HPAI) A(H5) viruses of clade 2.3.4.4b caused the worst ever observed epidemics in birds during the 2020/21, 2021/22 and ongoing 2022/23 epidemic years in Europe. They caused 14,325 reported outbreaks with devastating consequences for farmed poultry, resulting in the culling of roughly 96 million farmed birds in Europe. Some remarkable changes of epidemiology have been observed over the last years in relation to circulating HPAI viruses of the H5 subtype.

Highly pathogenic avian influenza epidemics in Europe used to be a seasonal phenomenon associated with migratory waterfowl returning to the overwintering sites in Europe in autumn, causing spill-over to farmed birds and outbreaks in poultry in the autumn/winter with some limited circulation in the spring. In 2021, for the first time, a few HPAI virus findings in wild birds continued over the summer in some breeding areas in northern Europe [1]. They were followed in 2022 by large outbreaks in the winter and spring in wild birds, that continued during the summer months with mass mortality in colony-breeding seabirds in several European countries.

In addition to the change in seasonality there was notable large global expansion and in 2020, A(H5) HPAI viruses of clade 2.3.4.4b spread west to east along migratory bird routes and were detected in south-east Asia [2]. In 2020/21 and 2021/22, a Europe-wide extension of the affected geographical areas was observed, including in countries that had never detected HPAI viruses before, such as Iceland, Norway (including Svalbard), and Greenland. An east to west spread via Iceland and Greenland was observed for the first time with A(H5N1) virus introductions from Europe to North America, progressing rapidly across large areas of Canada and the United States (US). Similar to the European situation, massive outbreaks in wild birds and spill-over to poultry farms causing extensive losses were observed. A north to south spread from Europe and North America to Africa and Central and South America, respectively, occurred as well [3]. The rapid expansion of A(H5N1) viruses following their introduction during the autumn bird migration in Central and South America down to the southern tip of Chile is unprecedented.

The rapid spread of A(H5N1) viruses to many previously unaffected areas globally and their successful persistence during the summer months was likely facilitated by the ongoing evolution and reassortment with local low pathogenic avian (LPAI) viruses, leading to their adaptation to newly or previously very rarely affected wild bird species such as barnacle geese or sea birds. This impactfully introduced HPAI viruses into local wild bird populations representing a threat for endangered bird species. Massive die-off events were observed in colony-breeding seabirds in Europe [4]. In South America, where a completely naïve wild bird population was affected by influenza A(H5N1), the virus caused a die-off of more than 40% of the pelican population in Chile and Peru that were classified near threatened already before the HPAI virus introduction, while affecting also other wild bird populations [5]. Should the expansion continue, also penguin colonies and other endangered species in the Antarctic may be at high risk. In addition, environmental factors and conditions might force wild bird species to leave their usual habitat leading to new interactions with different bird species. Such conditions would favour further viral evolution and reassortment processes as well as adaptation to different species.

Several transmission events to various free-living and captive mammalian species have been reported from many countries globally [6]. Transmissions to mammals have been mostly identified in individual animals, mainly wild carnivores preying on infected sick or dead wild birds or likely exposed to a highly contaminated environment. A variety of marine mammal species have also been found infected in Europe as well as in the Americas along the Atlantic and Pacific coasts. In addition, mass mortality events linked to HPAI virus infections with potential transmission between the same mammal species have been described among seals, among farmed minks and among sea lions [7-10].

Reports of infected dead or sick wild birds and terrestrial wild mammals (foxes) in urban or peri-urban areas have contributed to an increased environmental viral contamination and increased the risk of spill-over events to pet animals. Mammals infected with HPAI A(H5N1) virus clade 2.3.4.4b have been found ill or dead with severe neurological and, to a lower extend, with respiratory signs. High viral load was detected in the brain tissue of many of those infected mammals in line with the neurological clinical signs.

A few A(H5N1) virus clade 2.3.4.4b detections in humans without or with very mild symptoms such as fatigue, have been reported from the United Kingdom [11], Spain [12] and the US [13] related to exposure to infected birds or culling activities. In all these instances, a low number of viral copies was detected in the specimens taken from the people and no full-length genomic sequence data could be generated. Together with the lack of respiratory or other relevant symptoms as well as missing seroreactive antibodies to A(H5) viruses providing serological evidence, this indicated rather transient mucosal contamination of the nose instead of productive or systemic infection [11-13]. Therefore, the Spanish authorities consider these detection as non-cases. However, human influenza A(H5N1) virus infections caused severe disease in people in Ecuador and Vietnam (unknown clade) and fatal cases were reported in Chile and China, following exposure to sick or dead backyard poultry or to contaminated environment [14-16].

Similar to the severe acute respiratory syndrome coronavirus 2 (SARS-CoV-2) situation, the widespread circulation of HPAI viruses has been driving the high speed diversification for HPAI viruses with the emergence of different genotypes and reassortment events with local LPAI viruses independently in different geographical regions after introduction.

Although currently circulating avian influenza viruses retain a preference for avian-type receptors, different mutations associated with transmission to and pathogenicity in mammals have been observed. These mutations were detected sporadically in infected wild and domestic birds and more often emerged upon transmission events to mammals [6].

Highly pathogenic avian influenza viruses have become persistent in many regions globally, including Europe, and represent a constant threat for wild birds and mammals, the farming sector as well as public health. Ongoing evolutionary processes with ongoing virus circulation, diversification, and transmission to mammals, will continue and might increase mammalian-adaptive mutations associated with enhanced risk of transmission to and between mammals, including humans, which needs to be monitored across the kingdom of animals also in the light of the risk of a new pandemic.

So far only dead or sick wild animals have been tested for A(H5) viruses; information about asymptomatic infections in wild mammals, however, is lacking due to the absence of systematic monitoring. Studies in different wild and domesticated animal species, particularly in terrestrial and marine mammals, need to be conducted, including serological and molecular investigations, to identify the magnitude of the spread.

Further studies are needed to link clinical signs in infected animals with different organ affection over time, and to investigate the potential routes of infection in animals but also humans, duration of viral shedding in different animal species and optimal testing scheme for monitoring purposes to better assess the risk for human infection and propose prevention and mitigation measures.

Introduction of avian influenza viruses into mammalian populations is of concern because of the potential increased risk of reassortment between different influenza viruses, adaptation to and spread between mammals.

The risk for human health is currently limited to the animal-human interface when people are in contact with infected animals (birds or mammals) or contaminated environment. Introduction of the virus into urban areas with exposure of pets to infected birds increases this risk to humans and pet holders in affected areas should prevent contact between pets and sick or dead wild birds or mammals. Also, awareness of veterinarians is important to test pets (e.g. if respiratory or neurological signs appear after possible virus exposure) for avian influenza and identify infected animals as early as possible.

Despite the massive occurrence of wild bird mortality, poultry outbreaks and spill-over to wild carnivore species including environmental contamination and human exposure during these events, only sporadic human infections have been reported globally. In the latest instances, severe disease was observed following exposure to infected poultry in backyard settings, as well as to a contaminated environment or infected animals [15-17]. Mild infections might have been missed and serological data from exposed people are needed to better assess the situation while information on population immunity in humans is lacking.

Circulating A(H5N1) viruses have been assessed in the World Health Organization vaccine composition meeting to be antigenically similar to the recently developed A(H5) candidate vaccine viruses for pandemic preparedness considered for use in humans [18]. Some mutations associated with resistance to antivirals have been observed, but antivirals remain the main treatment option for humans.

To tackle the threat of avian influenza, a One Health approach is needed through rapid sharing of information about outbreaks, provision of sequence data and reference viruses, and close collaboration between the different sectors locally and globally. Communication campaigns may help to increase awareness in the population and recognise avian influenza viruses as a threat to animal and human health, in order to reduce the risk of contact with potentially infected animals.

With the ongoing global presence of A(H5) HPAI viruses, further sporadic spill-over events to humans cannot be excluded. It is crucial to identify such events as early as possible to timely initiate appropriate measures across all sectors.
